# Cognition in Climate Change: Is It Just a Matter of Time?

**DOI:** 10.1002/wcs.70014

**Published:** 2025-10-02

**Authors:** Massimo Bertoli, Martina De Cesaris, Sofia Bonventre, Marcella Brunetti

**Affiliations:** ^1^ Department of Neuroscience, Imaging and Clinical Sciences University ‘G. d'Annunzio’ of Chieti‐Pescara Chieti Italy

**Keywords:** climate change, cognition, eco‐anxiety, time cognition

## Abstract

Climate change (CC) is a global phenomenon characterized by long‐term shifts in temperatures and weather patterns. Aside from natural causes, we have been facing a full‐blown climate crisis primarily driven by human activity, leading to increasingly frequent and extreme weather events that put a strain on people's mental capacities. Addressing CC necessitates a temporal perspective as both causes and potential solutions extend beyond the present. However, despite being a significant challenge for humanity, CC is often considered temporally distant, leading to abstract thinking and reduced urgency for action. Considering the diverse dimensions that concur to define CC, this review will explore the link between CC and time cognition, building on insights from cognitive sciences. Upon considering the tangible effects of the anthropogenic CC (Changing Place), we argue that change in the social construction of time is inherent to CC and drifts to the point of affecting psychological well‐being (Changing Time). Moreover, considering that time is central to cognition and interlinked with several cognitive functions, we will consider the literature investigating the impact of CC‐related eco‐anxiety on cognitive abilities within the framework of time cognition. Furthermore, we assess how eco‐anxiety and time cognition interact, potentially serving as markers of mental well‐being (Changing Thoughts). By framing CC within the realm of time cognition, we offer an interdisciplinary perspective on cognition and well‐being, advocating for the integration of cognitive science into climate adaptation and mitigation efforts to foster more effective, psychologically sustainable long‐term climate strategies (Changing Future).

This article is categorized under:

Neuroscience > Cognition

## Graphical abstract

Climate change affects both the environment and human cognition, particularly by influencing how we perceive and process time. This review explores how cognitive science can inform climate adaptation and mitigation strategies through an interdisciplinary perspective.

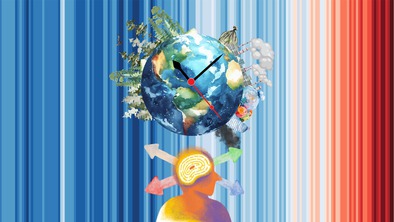

## 1 Introduction

**FIGURE 1:**
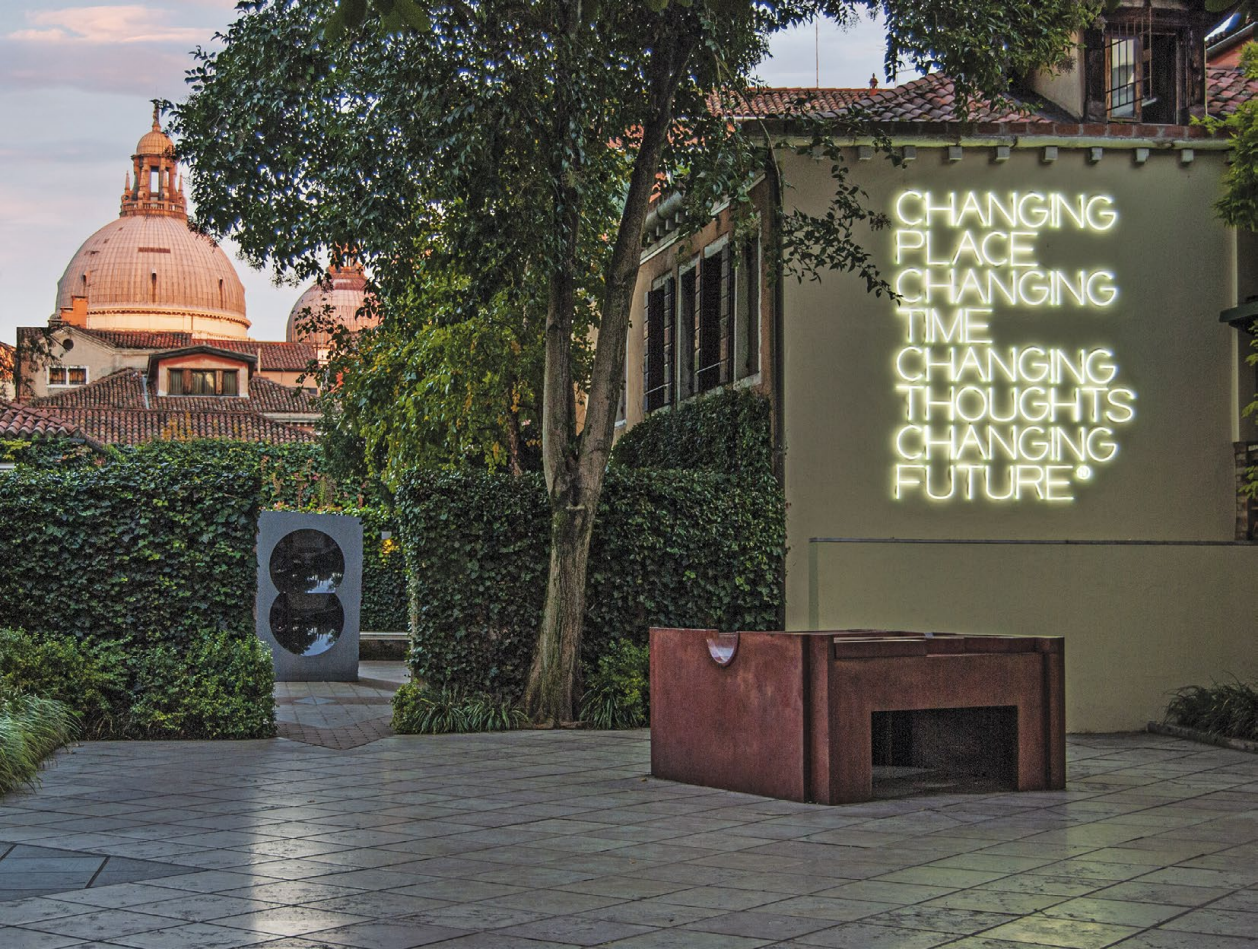
Maurizio Nannucci, Changing Place, Changing Time, Changing Thoughts, Changing Future, 2003. Neon tubes. Private Collection, Stetten, Germany. Long‐term loan to the Peggy Guggenheim Collection. Courtesy of Maurizio Nannucci.

“Changing Place, Changing Time, Changing Thoughts, Changing Future” (Figure 1) by the Italian artist Maurizio Nannucci is the title of an artwork displayed on the walls of *Palazzo Venier dei Leoni* in Venice, Italy. Simple in its realization, it still speaks to viewers' conscience by directing their thoughts toward four instances of change: place, time, thoughts, and future.

Anyone who sets foot in Venice will realize how this city fully exposes the precarious balance on which its fragile beauty rests—a balance threatened by change, particularly climate change (CC).

CC refers to persistent alterations in the statistical properties of the climate system—including changes in mean temperature, precipitation, and variability—occurring over decades or longer. These changes may result from natural processes or human activities (Intergovernmental Panel on Climate Change (IPCC) 2023; WMO 2024).

Beyond being an environmental and political issue, its extension over a wide time frame makes it relevant to human psychology and cognition, particularly in how we process change and anticipate future outcomes.

**FIGURE 2:**
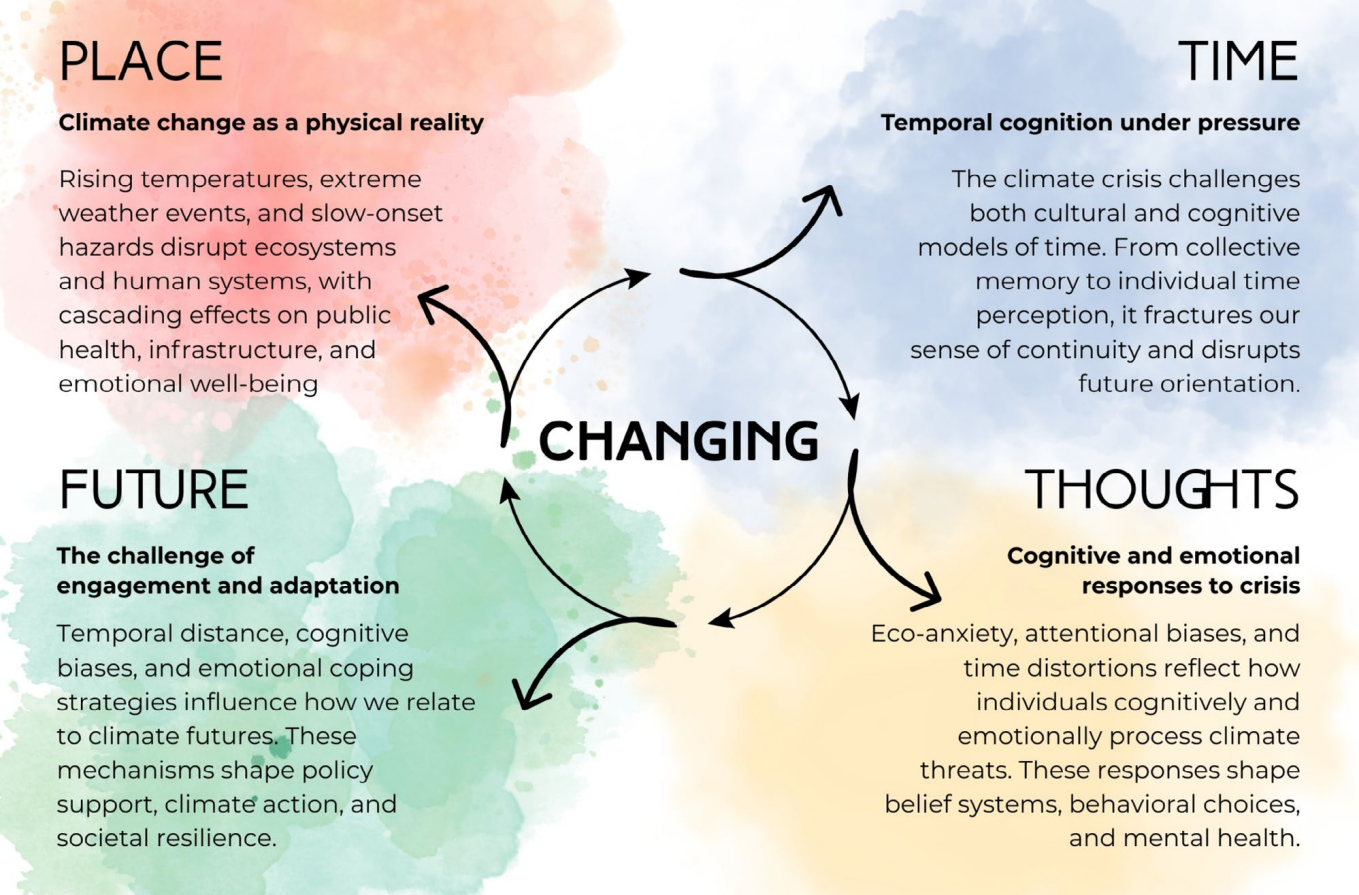
Conceptual overview of the manuscript structure. Climate change initiates transformations across four interconnected domains: Place (physical reality), Time (temporal cognition), Thoughts (cognitive and emotional responses), and Future (engagement and adaptation). These domains dynamically influence one another in a circular process of change, shaping how individuals and societies perceive, experience, and act on the climate crisis.

This review mirrors the structure of Nannucci's work, exploiting the concepts of time and change as threads to explore how cognitive mechanisms shape psycho‐behavioral responses to CC and inform adaptation and mitigation strategies (Figure 2).

We begin with *Changing Place*, which narratively brings together scientific evidence from complementary disciplines to establish the reality of anthropogenic CC and its existential threat to human and ecological systems. From this foundation, *Changing Time* provides an interdisciplinary perspective to frame CC as a potential cause of disruption of the temporal experience. The fracture of our sense of continuity, the sense of urgency, and future‐oriented cognition pave the way to emotional consequences and psychological well‐being, ultimately leading to states such as eco‐anxiety. Building on these insights, *Changing Thoughts* investigates how climate change‐related psychological manifestations and cognitive abilities shape beliefs, behaviors, and mental health outcomes. We therefore aim to corroborate the hypothesis that time perception, central to cognition and interlinked with several other cognitive functions, may serve as a valuable domain to detect changes relevant to psychological well‐being in the context of CC. Finally, *Changing Future* connects these psychological and cognitive mechanisms to the broader societal challenge of climate adaptation and the need to find mitigation strategies. Considering recent evidence that questions the efficacy of communication emphasizing the temporal distance of CC, we advocate for a prominent role of cognitive sciences within an interdisciplinary discourse aimed at adequately handling information regarding CC, arguing that climate policy must integrate temporal cognition, intergenerational equity, and emotional engagement to be sustainable and fair.

## 2 Changing Place

### 2.1 Climate Evidence: Rising Temperatures and Climatic Threats

To understand how CC affects human experience, we begin with its physical reality. CC is not only a scientific abstraction or a political issue; it is a material phenomenon that is reshaping the environments in which people live. From this starting point, we explore how environmental changes in the spaces people inhabit shape time perception, emotional response, and future‐oriented behavior.

Among the various indicators used to monitor CC, global surface temperature is considered the most fundamental and integrative measure. It captures the net effect of greenhouse gas accumulation in the atmosphere and serves as the primary benchmark for identifying long‐term climate trends (Cheng et al. 2019; Forster et al. 2025; Intergovernmental Panel on Climate Change (IPCC) 2023). While natural causes have always been implicated in the CC (e.g., variations of Earth's orbit, exposure to solar energy [Lourens 2021], and volcanic eruptions [Robock 1991]), there is a unanimous consensus among climate scientists that the current warming trend over the past two centuries is primarily driven by human activities.

Technological advances since the 1970s have enabled detailed reconstructions of past climates using paleoclimate evidence (e.g., ice cores, tree rings, and satellite data). These highlight that the current trend is unprecedented in both speed and magnitude, occurring 10× faster than past natural changes (Gaffney and Steffen 2017). Since the mid‐1800s, the development of economies based on the exploitation of fossil fuels such as coal, oil, and gas has led to an intensification in the production of greenhouse gases (mainly carbon dioxide, methane, and nitrous oxide) that have wrapped the Earth's atmosphere by sealing solar energy as heat (Ortiz and Jackson 2022), mostly stored in the oceans' waters (Cheng et al. 2025).

**FIGURE 3:**
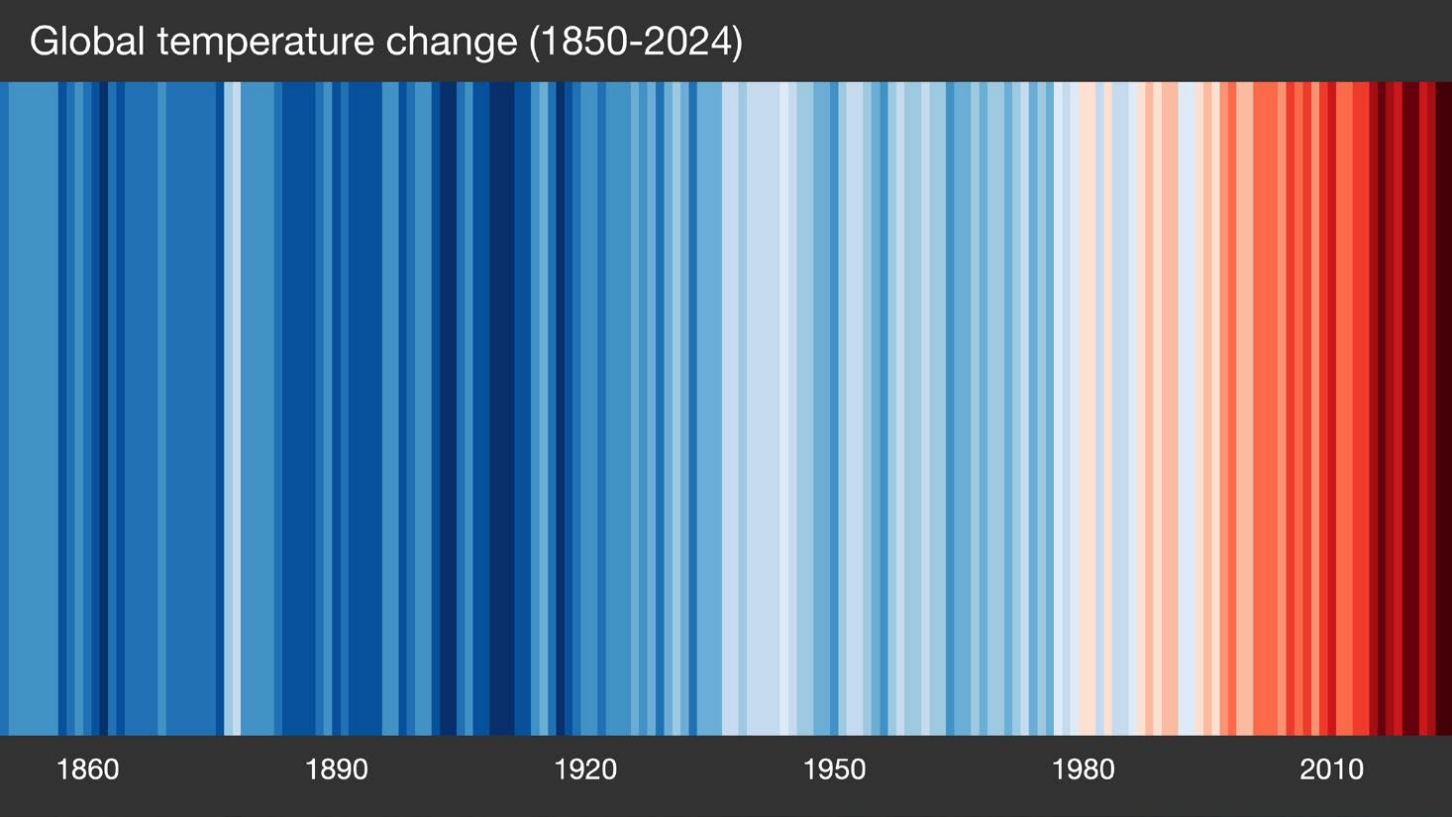
“Warning Stripes” by Ed Hawkins (University of Reading) depicts the global temperature change between 1850 and 2024 (available at showyourstripes.info). Each stripe represents a single year or month, with blue or red color indicating a negative or positive anomaly, respectively. Global average temperature change is calculated over the reference period 1961–2010 with the color scale ranging between +/− 0.9°C.

Yet, global temperatures continue to rise, with 2023 recorded as the hottest year to date (1.48°C above pre‐industrial levels), followed by 2024 at 1.55°C (±0.13) (WMO 2024, 2025) (Figure 3).

These changes are far from trivial, given the significant mortality linked to temperature extremes. In this regard, a recent study conducted by Masselot et al. (2025), which analyzed data from 854 European cities, projects that heat‐related mortality could increase by up to 50% by the end of the century—resulting in an estimated 2 million cumulative deaths—even under scenarios that include adaptation measures. The highest vulnerability is projected for Southern European countries, particularly those in the Mediterranean region, which are expected to experience the most severe temperature increases.

In the last “State of the Global Climate” Report filed by the World Meteorological Organization (WMO 2024), according to key indicators (global average temperature, concentration of greenhouse gases, ocean heat, sea‐level rise), the current situation is defined as a “climate crisis,” marking 2023 as the “black year” for the climate given that all previous records have been overcome, ultimately resulting in extreme weather events (e.g., extreme heatwaves, floods, wildfires, and intense droughts).

In fact, while rising temperatures are the primary and most robust indicator of anthropogenic CC, they give rise to a cascade of environmental disruptions. These include both acute extreme events and slow‐onset processes (e.g., sea‐level rise, soil erosion, and desertification), all increasingly linked to human activity (Intergovernmental Panel on Climate Change (IPCC) 2023). These consequences are not isolated but often interact in compound and cumulative ways, intensifying risks to ecosystems, infrastructure, and human well‐being.

For instance, floods and hurricanes have increased in both frequency and intensity, contributing to mass displacement, food insecurity, and infrastructure collapse (Rocque et al. 2021). In parallel, droughts and desertification accelerate land degradation, reduce crop yields, and heighten water scarcity, especially in low‐income regions with limited adaptive capacity (Gawhare et al. 2023; Sabola 2024).

Aside from breaking records, extreme events cause widespread damage in several aspects of life, including physical (Rocque et al. 2021) and mental health (Clayton 2021). This wider impact is captured by the Sustainable Development Goals (SDGs) adopted by the member countries of the United Nations for peace and prosperity for people and the planet (UN 2015). The SDG Progress Report highlights that in 2024 alone, the compounded effect of conflicts and environmental shocks (Hegre et al. 2016) has affected the economic conditions of a substantial portion of the world's population (“End poverty in all its forms everywhere,” SDG 1). One of the most significant consequences is reflected in agricultural productivity and production of food resources that have significantly raised global hunger levels (“End hunger, achieve food security and improved nutrition and promote sustainable agriculture,” SDG 2). For example, the alteration of temperature and precipitation patterns mainly affects the productivity of rain‐fed agricultures (e.g., Southern Africa [Sabola 2024] and South Asia [Gawhare et al. 2023; Mann 2021]) with variable impacts on crop yields across regions and crop types. Furthermore, these effects interact disproportionately at a socioeconomic level with vulnerable developing countries, typically relying on agriculture and with limited capacity to adapt to changing conditions, thus leading to a protracted displacement of people (“Reduce inequality within and among countries,” SDG 10) (Askland et al. 2022).

In sum, the material reality of CC directly threatens ecosystems and social systems alike. Yet despite mounting evidence, CC often fails to translate into coherent perception or sustained action. One reason may lie in its disruption of not only physical systems, but also our temporal experience—fracturing the continuity between past, present, and future. In the next section, we explore how CC challenges our conception and experience of time and its implications for cognition and action.

## 3 Changing Time

Having examined the material and environmental dimensions of CC, we now turn to its temporal implications. We propose that anthropogenic CC—and our responses to it—are part of a dynamic process of (un)tuning between sociocultural and natural rhythms. In particular, we explore how the climate crisis challenges traditional conceptions of time, proposing that the subjective experience of time interacts with temporal cognition to give rise to novel experiences of disorientation, disconnection, and urgency—the hallmarks of the “changing times.”

This untuning becomes evident in how CC challenges our timelines: we are asked to act NOW on causes rooted in the PAST to avoid consequences that may only materialize in the FUTURE. Such time‐bounded urgency is reflected in long‐term mitigation strategies for lowering greenhouse gas emissions like the Paris Agreement (COP21), which aims to limit warming to 1.5°C (2.7 F). Nonetheless, approaching tipping points, that is, critical thresholds beyond which the effects of CC are irreversible (McKay et al. 2022), and the lagged effects of greenhouse gas concentration (Armour and Roe 2011) reveal that the consequences of today's inaction will unfold over centuries.

In what follows, we explore how climate change challenges dominant conceptions of time. The following paragraphs examine the historical evolution of the concept of time (Section 3.1), highlighting how individuals and societies perceive and use temporal information (Section 3.2), and finally exploring its connection to CC (Section 3.3).

### 3.1 The Evolution of Temporal Meaning: From Objective to Subjective Time

The most challenging dimension of CC, both for individuals and society, is its extension in time, particularly its extension in the future (Pahl et al. 2014).

The meaning of time has evolved historically, from cyclical natural rhythms (sun, moon phases, and seasons) to linear and abstract forms shaped by scientific and technological advancements. In Western societies, industrialization and the establishment of Newtonian physics contributed to giving rise to a view of time as measurable and actionable, thus emphasizing values such as punctuality, efficiency, and time optimization (Reisch 2001). In contrast, phenomenological and existential philosophical traditions of the 20th century reframed time as a unified flow, subjectively experienced and constitutive of phenomena and human existence itself (Gallagher 2013; Heidegger 1962; Husserl and Brough 1991). This tension between objective clock‐time and subjective lived‐time is crucial in understanding how modern societies struggle to engage with long‐term threats like CC.

### 3.2 Navigating Subjective Time: Memory, Imagination and Climate Crisis

#### 3.2.1 Personal Future and Temporal Self‐Continuity

Alongside the objective and measurable dimension of time, subjective time accounts for the complexities of human temporal experience (Arstila 2016). While the scientific understanding of time is helpful for its measurement, subjective time permeates the core of unique, first‐person experiences, revealing that time is not strictly linear and objective but shaped by factors rooted in emotions, cognitive processes, and contextual elements (Davidsen 2013).

A practical example of this is the ability to project oneself in time remembering past events or simulating future ones; that is mental time travel (MTT) (D'Argembeau and Van der Linden 2006).

This ability is bidirectionally linked to the attitude with which one both understands and relates to different temporal dimensions, that is, time perspective (Zimbardo and Boyd 1999). An effective interaction between time perspective and MTT has an evolutionary function, as it allows planning and adaptation to the environment by retrieving elements of past experiences to be integrated to simulate future events, ultimately increasing the sense of control and self‐continuity in time. Theoretical work argues that, while some animals display elements ascribable to episodic memory and future‐oriented cognitive abilities, MTT is uniquely human, reflecting the complexity of time cognition and its interaction with cultural features, primarily language (Martin‐Ordas 2020).

MTT involves episodic memories related to specific personal events, as well as the ability to envision the future and simulate future scenarios through episodic future thinking (Klein 2013; Szpunar 2010). Research in cognitive neuroscience supports this continuity, showing that mental time travel relies on shared cognitive and neural systems for both past and future simulation (Schacter et al. 2012, 2017; Schacter and Addis 2007). Functional neuroimaging studies have identified a distributed neural network activated both when imagining the future and when recollecting the past. This network includes the prefrontal cortex, medial temporal regions, and parietal areas, which together support self‐referential integration of past experiences and future simulations through the flexible allocation of attentional resources (Smallwood and Schooler 2015; Suddendorf and Corballis 2007).

#### 3.2.2 Shared Futures: Collecting Memories and Imagine Climate

MTT ability extends beyond individual experiences to how a group of people constructs and shares its history and how they imagine their future—a distinct collective process, not merely a sum of individual MTT abilities (Szpunar and Szpunar 2015). Evidence from patients with hippocampal damage supports this distinction: individual MTT may be impaired, while collective MTT remains intact (Mullally and Maguire 2014; Szpunar et al. 2014, 2016). Moreover, while individual MTT is rich in sensory and personal details, collective MTT is an interactive process where shared narrative and systems of values forge collective history and future orientation. It also involves inherently social processes of collaborative inhibition and/or facilitation (Hirst and Echterhoff 2012), intergenerational transmission of narratives, synchronized intentions, and responsibility for planning and imagining a collective future (Michaelian and Sutton 2019).

Divergence between individual and collective MTT is also reflected in cultural time perspectives. For example, Western North American and European individuals show a positivity bias for their personal future but pessimism for their group's future, regardless of background, age, gender, political view, and distance of imagined future (Shrikanth et al. 2018; Shrikanth and Szpunar 2021).

By contrast, cross‐cultural comparisons suggest that this pattern is not universal. A recent comparison of Western and Eastern Chinese participants revealed that the Chinese individuals exhibit a more positive bias toward both past and future events, possibly reflecting a culturally embedded vision of change as a natural process and shaped by specific socio‐political drivers.

Notably, in the collective future domain, pessimism remains stable across age groups in Western cultures. Conversely, Chinese older adults tend to maintain a positive stance, while younger Chinese adults align with their Western counterparts (Yao et al. 2025). These findings suggest that collective future thought is shaped by culture and socio‐economic factors.

Yet globally, younger generations remain the most vulnerable to climate‐related uncertainty (Kashima et al. 2025). In this picture, narratives driven by media prioritizing negative information over positive or uncertain information may reinforce a dystopic future perpetuating a cycle of climate pessimism and negative expectations about the future. However, cultural differences show that alternative narratives about the future are possible. Exploring how social and ecological rhythms are co‐constructed (Wood 2008) may help shed light on unprecedented phenomena of collective disorientation. Thus, in a world shaped by CC, the disruption of these collective and ecological rhythms may in turn lead to an altered experience of time.

### 3.3 Climate Crisis and the Breakdown of Temporal Continuity

Disruptions in subjective time experience, or temporal disintegration, affect both individual and collective psychological well‐being (Melges 1982). Temporal disintegration refers to a discontinuity in the flow of time, impairing the ability to structure experiences within a unified temporal framework (Moskalewicz and Schwartz 2020). While it has been observed in psychopathological conditions such as PTSD, depression, and anxiety (Fuchs 2020), its broader implications have become more visible in collective contexts (e.g., global crises and environmental degradation) and are enhanced by media‐driven hyperconnectivity (Alison Holman et al. 2022; Silver et al. 2002).

As the anthropologist Marc Augé argues, contemporary society faces a diminished ability to navigate time, shaped by global phenomena such as CC, technological pervasiveness, and the erosion of historical continuity (Marc Augé 2010). The resulting loss of a cohesive collective narrative leaves societies fragmented, struggling to project a meaningful future beyond a present dominated by uncertainty and existential threats (M. Augé 2003). This shifting temporal framework serves as the backdrop for what Augé calls “new fears”—emotional states of restlessness and uncertainty that arise from an altered relationship with history and the future (Marc Augé 2013). CC exemplifies the disruption of temporal continuity by severing ties between past, present, and future. While media‐driven narratives of catastrophe and urgency have enabled unprecedented access to information, on the one hand, they have also contributed to time compression—a paradoxical acceleration that fosters a widespread sense of time scarcity, intensifies helplessness, and undermines long‐term planning (Höijer 2010; Rudd 2019; Ruiu 2021; Thompson et al. 2019; Woodward and Jones 2008; Ytre‐Arne et al. 2020).

Unlike past fears, which were anchored in defined temporal or geographical contexts, new fears arise from global crises that transcend traditional boundaries, making them diffuse and persistent. The perception of an accelerating degradation of the world as we know it fuels a collective disorientation, deepening the challenge of reconciling the present with an increasingly uncertain future. In this sense, CC represents a source of chronic stress, primarily due to the loss of environmental familiarity and the severing of ties with both personal and collective history, ultimately eroding the ability to envision a meaningful temporal framework. This growing emotional and cognitive burden marks the transition from temporal disruption to affective response. In the following section, we explore how altered time perception interacts with cognitive processes to give rise to climate‐related psychological phenomena such as eco‐anxiety.

## 4 Changing Thoughts

In the previous section, we explored how CC challenges both objective and subjective experiences of time, altering how individuals and societies relate to the past, present, and future. These temporal disruptions deeply influence how we think, feel, and act in the face of climate threats.

In this section, we investigate how cognitive processes and belief systems interact with climate‐related information (Section 4.1). We then explore how the emotional responses like eco‐anxiety shape mental representations of CC (Section 4.2). Finally, we examine the interplay between CC and time, from both a cognitive (Section 4.3) and affective perspective (Section 4.4).

### 4.1 Thinking Climate: Cognitive Mechanisms and Belief Systems

The emotional burden of CC demands significant cognitive engagement. Understanding the underlying cognitive mechanisms is crucial to explain why individuals differ in their responses, ranging from proactive engagement, heightened concern, and high levels of distress, skepticism, or denial.

Concerning attentional processes, Whitman et al. (2018) investigated how climate concern shapes visual attention using eye‐tracking methods. They showed that, compared to individuals with low concerns about climate‐related information, highly concerned individuals prioritized climate‐related words over neutral ones, suggesting an attentional bias similar to threat‐related biases observed in generalized anxiety disorder (Bar‐Haim et al. 2007). Expanding on this, Luo and Zhao performed a series of eye‐tracking experiments, focusing on the relationship between socio‐political motivation, visual attention, and perception of CC evidence (Luo and Zhao 2019). According to the Motivate Attention Framework (Luo and Zhao 2021), individuals would indeed allocate their attentional resources to climate change‐related information based on beliefs, political positions, and cognitive motivations that influence how the information is processed. In their study, participants were presented with graphs depicting global temperature historical trends: liberals attended more to the rising phase of the temperature curve indicative of CC before providing higher estimates of average global temperature. Conversely, conservatives focused more on the flatter phases, reporting lower average temperature estimates. Moreover, this trend was associated with the willingness to enact pro‐climate behaviors (such as signing a petition or making a monetary donation to the climate cause) but only in individuals with liberal orientations. Lastly, in a follow‐up study, the attentional focus was manipulated by color‐coding temperature trends (either highlighting in red the rising or flat trend). They found that color‐coding successfully oriented visual attention in both groups, with liberals more likely to engage in climate action when the rising trend was highlighted. However, conservatives became less likely to act when strong climate evidence was emphasized, suggesting that tailoring CC information to specific cognitive features can influence how that information is treated and acted upon (Luo and Zhao 2019).

These findings align with an integrative cognitive framework (Zhao and Luo 2021) advancing that climate‐related beliefs are not only the result of knowledge or of the gathering of objective data but can emerge from multiple cognitive biases—such as attentional, perceptual, and decision‐making processes—that need to be considered when dealing with mental representations of CC.

### 4.2 Eco‐Anxiety: A New Fear for a Threatened Future

In the realm of future‐oriented fears, the term “eco‐anxiety” has been introduced to describe the experience of “heightened feeling of distress related to ecological crises, including anthropogenic climate change” (Pihkala 2020). This feeling could stem from direct exposure or anticipatory distress about CC intolerance to uncertainty about long‐term environmental stability exacerbated by media and social discourse (Castelnuovo et al. 2024; Clayton 2020).

Although most of the scientific literature concerning the effects of exposure to climate‐related extreme weather events originates from studies in Western countries (Coffey et al. 2021), systematic reviews indicate that individuals directly affected by climate‐related disasters experience heightened levels of psychological suffering, often leading to chronic distress and trauma‐related symptoms (Clayton 2021; Walinski et al. 2023). However, it is clear that these effects disproportionately affect vulnerable populations, including young people, individuals with pre‐existing mental health conditions, and those living in high‐risk areas for extreme weather events (Ngcamu 2023).

In contrast, indirect exposure to CC can amplify eco‐anxiety related feelings, particularly among younger generations (Hickman 2020). This aligns with findings from a large‐scale survey across 84 countries, which found that 59% of young respondents reported feeling extremely concerned about CC. At least 89% of them were moderately concerned, while 45% also reported significant impairment in daily functioning due to negative thoughts and emotions related to CC and insufficient institutional response (Hickman et al. 2021).

#### 4.2.1 Between Concern and Collapse: Cognitive Regulation of Emotional Distress and Behavioral Responses

Eco‐anxiety encompasses a broad range of emotions, from concern to existential dread and grief from environmental loss—the latter is known as solastalgia (Albrecht 2005). These emotions are shaped not only by individual traits but also by cultural narratives and psychological bonds to place, which can amplify risk perception and emotional distress or, conversely, act as barriers to coping behaviors (Bonaiuto et al. 2016; Ogunbode et al. 2022; Ojala et al. 2021).

Recent findings suggest that behavioral outcomes are not solely driven by the emotional burden of eco‐anxiety but are moderated by cognitive processes regulating attentional focus. In fact, a more variable attentional focus fluctuating between climate threats and other stimuli appears associated with higher levels of distress and lower engagement (Mathers‐Jones and Todd 2023), whereas a more stable attentional focus on climate‐related threats plays a crucial role in transforming climate concern into pro‐environmental action.

However, an excessive attentional focus may also lead to an overwhelming feeling, resulting in inaction rather than motivating pro‐climate behaviors. This evidence aligns with a cognitive and emotional profile ascribable to the broader spectrum of mood disorders, where heightened sensitivity to threats can lead to either proactive behavior or emotional paralysis. A resting state neuroimaging study on eco‐anxiety has pointed out the involvement of key brain areas involved in threat detection and behavioral response. Specifically, enhanced resting state functional connectivity between the midcingulate cortex, implicated in assessing and anticipating potential environmental threats, and the insular cortex, crucial in generating emotional and physiological responses, appears to be central to both anxiety disorders and eco‐anxiety (Carlson et al. 2024), supporting the claim that eco‐anxiety is not merely an emotional reaction but is embedded in our neurocognitive architecture in response to threats.

Far from being conceived as an undifferentiated affective phenomenon or a form of pathological anxiety, eco‐anxiety is better conceptualized as a form of practical anxiety emerging in response to real, ongoing, and scientifically substantiated threats. Unlike pathological forms of anxiety that can lead to avoidance, emotional numbing, and mental health deterioration (Amstadter 2007), practical eco‐anxiety supports the emerging of behaviors (e.g., information gathering, reflection, and active engagement) aimed at helping address the problem and fostering well‐being and motivating pro‐environmental behaviors (Kurth and Pihkala 2022). However, exaggerated or persistent eco‐anxiety, especially when compounded by uncertainty, perceived inefficacy, and institutional distrust, can result in maladaptive responses sharing psychological distress, disengagement, and avoidance behaviors (Crandon et al. 2024; van Valkengoed and Steg 2024).

Together with an increasing understanding of the affective experience of eco‐anxiety, deepening the cognitive and neural mechanisms appears critical for developing interventions that help individuals navigate climate distress while fostering resilience and pro‐environmental engagement.

### 4.3 Time Cognition and Emotional Distress in Eco‐Anxiety

Eco‐anxiety does not represent a clinical disorder per se. Rather, fluctuations between negative states and successful adaptation to climate anxiety are expected (Gago et al. 2024). Systematic reviews on the link between CC awareness and mental health (Gianfredi et al. 2024), as well as studies on CC and eco‐anxiety (Gago et al. 2024; Soutar et al. 2022) support the idea that heightened levels of stress about nature loss are closely associated with poor psychological outcomes (Pienkowski et al. 2024).

One of the most relevant experiential features of eco‐anxiety is the feeling of “running out of time.” This sense of temporal urgency heightens attentional bias toward climate‐related threats, reinforcing the cognitive load associated with distress. As individuals seek information to reduce uncertainty, they may paradoxically reinforce emotional exhaustion and face functional impairments in daily functioning such as changes of habits, lack of motivation, and social withdrawal (Nadarajah et al. 2022). In this context, time cognition—how people perceive, estimate, and simulate time—may play a central role in shaping both the emotional and behavioral responses observed in eco‐anxious individuals.

Nonetheless, while research on eco‐anxiety has primarily focused on its emotional and behavioral implications (Vlasceanu et al. 2024), emerging evidence suggests that eco‐anxiety may also alter time cognition itself. Specifically, chronic distress about CC may lead to distortions in attentional and memory processes, as these cognitive resources compete when processing climate‐related information. These distortions may impair psychological well‐being and adaptive engagement, underscoring the need to further investigate time cognition as a target for intervention, especially among vulnerable populations.

#### 4.3.1 Time Distortions: The Roles of Attention and Memory

Cognitive resources determine the ability to experience and estimate time, influencing the ability to process time durations, reconstruct temporal sequences, and anticipate future events to develop a coherent internal timeline and adapt behavior to multiple environmental changes (Koelewijn et al. 2010). This ability is defined as time perception and refers to time scales ranging from milliseconds to months and years (Grondin 2010) and relies on attentional control and memory retrieval (Buhusi and Meck 2005). The interaction between these two domains influences both prospective and retrospective timing paradigms, where individuals are respectively instructed to monitor the time or temporal information actively is implicitly encoded, as well as interval timing models that explain how the brain computes time durations.

#### 4.3.2 Attention Based Models

With respect to prospective time judgments, several models emphasize the role of attention while performing time estimates.

According to the Pacemaker‐Accumulator model (PAM), subjective estimation of time relies on an “internal clock” to measure objective time (Treisman 1963). In PAM, a sensory or cognitive event acts like a switch and starts the timing process involving a pacemaker that generates pulses at a steady rate. However, the speed of the pacemaker can vary based on arousal: higher levels of physiological activation increase the internal clock speed, letting more pulses accumulate and lead to perceived longer durations (Droit‐Volet and Meck 2007; Treisman 1963). On the contrary, lower levels of physiological activation slow down the internal clock, leading to perceived shorter duration (Wittmann and Paulus 2008). When timing stops, pulses are counted by the accumulator and used to estimate time.

An expansion of PAM is the Scalar Expectancy Theory (SET) (Gibbon 1977), which accounts for empirical evidence that time estimation errors grow linearly with the size of the interval timing estimate (Buhusi and Meck 2005; Grondin 2010), thus allowing a more flexible framework to explain variability in time perception. The model envisages three stages: internal clock, memory, and decision. During the first stage, time pulses are stored in the accumulator, similarly to PAM. The pulses are then accumulated in a working memory system, stored as a reference in a long‐term representation of the experienced number of pulses, and used to perform time estimates during the last decision stage.

The Attentional Gate Model (AGM) extends the SET by incorporating an attentional gate over temporal processing involved in the storing of the pulses (Zakay and Block 1996). According to the AGM, the allocation of attentional resources to timing would allow the gate to open wider, enabling more pulses to be transferred to the accumulator, resulting in more accurate temporal judgments and longer perceived duration (Coull et al. 2004). Additionally, predictive timing ability benefits from the allocation of attentional resources impacting accuracy (Nobre and Van Ede 2017) through increased sensitivity to expected temporal patterns, with the goal of anticipating adaptive behaviors (Coull and Nobre 2008).

On the contrary, when attentional resources divert from timing or are contended by another task (Macar et al. 1994; Zakay and Block 1996), the attentional gate does not open fully, allowing fewer pulses to pass through. Hence, a smaller pulse count in the accumulator (working memory) than the one in reference (long‐term memory) yields an underestimation of perceived duration.

Finally, Striatal Beat Frequency (SBF) theory (Matell and Meck 2004) provides a neurobiological extension of cognitive models of time perception, filling the gap in how time estimation is implemented at the brain level. SBF proposes that interval timing is realized through oscillatory activity in cortico‐striatal circuits, basal ganglia in particular (Meck 1996). This oscillatory pattern would represent the pacemaker while the striatum would serve as an accumulator that encodes time according to the convergence of rhythmic neural signaling. Internal clock cortico‐striatal circuits would be regulated by dopaminergic activity (Allman and Meck 2012; Meck 1996). Higher dopamine levels accelerate the speed of the internal clock, leading to overestimation of time while lower levels would slow the internal clock, accounting for time underestimation typically observed in clinical dopamine‐related conditions like Parkinson's disease. Moreover, dopamine would regulate precision temporal discrimination by adjusting timing prediction through interval encoding and reinforcement learning (Matell and Meck 2004).

#### 4.3.3 Memory Based Models

A different explanation for time perception ability, specifically concerning retrospective timing, comes from Zakay's Contingency Model (Zakay 1993), an extent of a previous Contextual‐Change model (Block and Reed 1978) which suggested that the estimated duration of an interval is a function of the number and richness of encoded memories of external (environmental) and internal (e.g., cognitive strategies and arousal) contextual changes. The Contingency Model relies on different mnestic abilities serving encoding and retrieving processes to track time. It posits that the more single events are memorized in relation to a specific time interval, the longer the interval will be perceived (Buhusi and Meck 2005; Eichenbaum 2013).

Nevertheless, it has been highlighted that time perception does not rely on a single process but results from the dynamic interplay between attention and memory depending on task demands, cognitive resources, and contextual features (Block and Gruber 2014): an Attentional‐Gate model could be more suitable to account for prospective time estimation depending on real‐time attentional monitoring, while a Contextual‐Change model could be needed to account for retrospective judgments where individuals reconstruct time based on encoded contextual details (Block 2003). Prospective time estimation (e.g., actively tracking time) aligns with attentional models (e.g., PAM, SET, and AGM). Retrospective time estimation (e.g., recalling past durations) aligns with memory‐based models (e.g., CCM and Contingency Model). Emotion and arousal further modulate both processes, altering perceived duration through interactions with attentional engagement, memory encoding, and pacemaker speed.

### 4.4 From Emotion to Cognition: Revisiting Time Distortions in Eco‐Anxiety and Affective Disorders

Given that affective disorders closely related to eco‐anxiety, such as anxiety and depression, are known to systematically alter time perception, it is plausible that eco‐anxiety shares a similar cognitive architecture. In mood disorders, time perception is often disrupted by distortions that shape temporal experiences. Anxiety disorders are characterized by prolonged states of future‐oriented arousal in response to uncertain threats (Droit‐Volet and Meck 2007; Lang et al. 2000), leading to temporal distortions due to suboptimal allocation of cognitive resources.

This distortion primarily manifests as attentional impairments, where a disproportionate allocation of cognitive resources to threat detection compromises the ability to track time accurately. Experimental evidence supports this claim: Mioni et al. (2016) demonstrated that anxious individuals under‐reproduced time intervals when performing a prospective time reproduction task. According to pacemaker‐accumulator models, this suggests that anxiety accelerates the internal clock, leading to the subjective underestimation of elapsed time. These results likely reflect impaired attentional and working memory processes involved in storing and recalling the temporal interval. Consistently, the hallmarks of anxiety—increased distractibility and difficulty in sustaining focus—support the observation that anxiety induces a narrower focus of attention toward threats at the expense of other cognitive processes such as time tracking and executive control (Bishop 2009).

Conversely, depressive states are associated with hypo‐arousal and lead to an overproduction of time intervals during a time production task (Mioni et al. 2016). Unlike anxiety, which predominantly affects attentional mechanisms, depression‐related distortions appear to stem from alterations in the internal clock mechanism, as evidenced by slowed time discrimination in mild depression (Msetfi et al. 2012). This is in line with cognitive theories of depression that account for a general slowed cognition, reduced physiological arousal, and impaired executive functioning as core features of the disorder (Steffens et al. 2022; Villalobos et al. 2021). Although not directly predictive of temporal distortions, the impairments in executive functioning observed in terms of cognitive shifting and inhibitory control align with literature indicating a weakened attentional control and cognitive rigidity as implicated in time distortions (Eysenck et al. 2007).

The memory bias hypothesis offers an additional explanation for timing distortion in anxiety, complementing the alteration of prospective time mechanisms. Within the Contextual Change Model (Block and Reed 1978), Liu and Li (2024) investigated the role of memory encoding and retrieval in retrospective time estimation among individuals with high state anxiety compared to individuals with low state anxiety. They reported that high state anxious participants estimated past time intervals to be significantly longer than low‐anxiety individuals, a distortion explained by the tendency to recall more negative contextual details. The role of negative time perspective, both in terms of past and future orientation, further supports temporal distortions in anxiety (Åström et al. 2019). In fact, anxious individuals exhibit a systematic negativity bias toward past and future orientations, with a negative past time perspective strongly predicting rumination and repetitive negative thinking, similar to depressive states, while a negative future time perspective is more associated with anticipatory anxiety.

Uncertainty and unpredictability also influence time perception in anxious individuals through cognitive and neurobiological pathways (Lake and LaBar 2011). Predictable threats and immediate fear tend to lead to time overestimation (Stetson et al. 2007), while uncertain threats and anxiety may result in time underestimation (Schmitz and Grillon 2012), reflecting the role of attentional control mechanisms and emotional processing biases in shaping the subjective experience of time. From a neurobiological perspective, uncertainty could result in a disruption of brain timing mechanisms. Research has highlighted how uncertainty amplifies activity in the amygdala and insula, brain structures involved in threat anticipation, namely anticipatory anxiety and emotional dysregulation (Sarinopoulos et al. 2010). Notably, these brain areas partially overlap with resting state neuroimaging evidence linking climate anxiety to functional activity within the insula cortex, part of a network involved in adaptation to aversive threats (Carlson et al. 2024). Additional evidence suggests that uncertainty disrupts the striatal beat frequency model of timing by inducing an inconsistent phase reset of cortical oscillation necessary for detecting and learning temporal patterns, also mimicking dopamine depletion (Allman and Meck 2012).

The relationship between time perception and climate‐related cognition is further supported by findings on procrastination and temporal discounting, a tendency to prefer immediate gratification over future benefits (Myerson and Green 1995; Zhang et al. 2019). People typically conceptualize the future within a timeframe of approximately 15 years (Tonn et al. 2006) while associating it with greater concern than the present moment (Shrikanth et al. 2018; Shrikanth and Szpunar 2021). This cognitive tendency becomes particularly relevant in the context of CC and decision‐making, where individuals struggle to balance short‐term personal concerns with long‐term environmental sustainability (Hardisty and Weber 2009). Besides, temporal distance also influences the richness of how we can simulate near or far future events. In fact, near future events elicit more episodic processing resulting in more vivid, detailed, and emotionally engaging simulations that require higher cognitive monitoring. Conversely, future but distant events would draw on generalized and more abstract semantic knowledge accounting for more abstract and decontextualized simulations (Colás‐Blanco et al. 2022). These findings suggest that the ability to simulate future events involving CC could be modulated by cognitive processes related to time perception. Overall, eco‐anxious individuals may experience time compression, retrospective distortions and hampered ability to envision a coherent future, all shaped by threat‐driven cognitive constraints.

The interdisciplinary dialogue thus indicates that time distortions that can be seen in eco‐anxiety are not mere perceptual anomalies, but deeply rooted in cognitive and emotional processes. Thus is crucial to consider how people process information about the climate crisis to enact more or less adaptive cognitive and behavioral strategies. By integrating these findings under the light of time cognition, we can outline a framework to deepen our understanding of eco‐anxiety. Considering its role in shaping emotional and behavioral responses to CC, an interdisciplinary understanding of how eco‐anxiety modulates cognition and behavior may help inform interventions aimed at fostering effective climate communication, adaptive engagement, and societal resilience to climate‐related distress. In the next section, we explore how these cognitive and emotional processes interact with broader motivational and social dynamics that shape our future responses—including engagement, mitigation, and climate policy.

## 5 Changing Future

Having examined the environmental, temporal, and cognitive‐emotional dimensions of the climate crisis, we now turn to how these mechanisms influence our relationship with the future. In this section, we explore how people perceive and interpret future risks (Section 5.1), make climate‐related decisions (Section 5.2), and engage in mitigation or adaptation efforts (Section 5.3). We argue that understanding temporal discounting, psychological distance, and collective coping strategies is essential to designing cognitively sustainable interventions (Section 5.4), ranging from personal behaviors to interdisciplinary climate policies (Section 5.5).

### 5.1 Psychological Distance of Climate Change: A Psychological Barrier to Action

CC is often perceived as distant—temporally, spatially, and socially—which hinders emotional engagement with the cause and introduces a psychological distance between urgency and motivation to act (McDonald et al. 2015; Spence et al. 2012). Perceived psychological distance is considered to be one of the main barriers to implementing pro‐climate behaviors and can be explained through the Construal Level Theory (CLT) (Trope and Liberman 2003, 2010). CLT describes how thinking, feeling, and acting on an event depend on the psychological distance and level of abstraction used with regard to events like CC that can be perceived as distant in terms of time (climate change is a future problem), space (it will affect other places), social proximity (it will affect others), and certainty (it is a hypothetical risk). Individuals would mentally construe different events in either an abstract or a concrete manner, depending on their perceived psychological distance. It follows that as distance increases, people use more relatively simple, abstract representations, whereas decreased distance would encourage people to construe events in more complex, concrete terms.

Psychological distance represents a crucial aspect in the relationship between CC and public perception since it influences risk assessment and public engagement in climate action. A study on a nationally representative sample of British individuals showed that CC is perceived both as distant and proximal, depending on specific dimensions of psychological distance. Notably, a reduced psychological distance was associated with greater concern and willingness to act against CC, especially when participants were made aware of the effects concerning developing countries (Spence et al. 2012). Similarly, Brügger et al. (2015) confirmed that CC feels like a more urgent topic when it is framed as close to the belonging group in time and space. However, when considering willingness to engage in mitigation vs. adaptation strategies, a more dynamic framing emerges: embracing mitigation measures appears more likely when CC is framed as distant, while adaptation measures are more likely to be adopted when spatiotemporal distance is reduced. This evidence seems to support the dominant narrative of CC being characterized primarily by great psychological distance, a characteristic that supports disengagement and inaction while also influencing policies of environmental awareness aimed at reducing the perceived distance.

However, the relationship between psychological distance reduction and pro‐environmental behavior engagement is not consistent across studies, suggesting that reducing psychological distance per se is not a sufficient tool to influence people's behavior (Maiella et al. 2020). Additionally, systematic studies challenge this idea by arguing that the concept of psychological distance itself is overestimated. Instead, people already perceive CC as imminent and affecting their communities (van Valkengoed et al. 2023). This situation, rather than being a paradox, reflects the complexity of individual responses to CC. The conceptual device of eco‐anxiety offers the potential to overcome this paradox by acknowledging how the distress that emerges from increased awareness of the climate crisis affects the behavioral response in a multifaceted manner (Dodds 2021). Proactive engagement and emotional disengagement are both possible outcomes of a temporal relationship with CC. However, rather than simply reducing perceived distance, we must also consider the emotional and cognitive consequences of climate awareness—particularly eco‐anxiety, which mediates engagement with the climate crisis (Crandon et al. 2024).

This nuanced relationship between psychological distance and engagement interacts with long‐term oriented temporal cognition and the tendency to prefer immediate gratification over future benefits known as temporal discounting. When declined to CC, this tendency results in lower rates of engagement in pro‐environmental behaviors (Hsia et al. 2025; Klein 2013; Polasky and Dampha 2021). However, it can be mitigated by leveraging episodic future thinking—the ability to imagine specific and personally relevant future scenarios. Indeed, by anchoring climate risks to specific scenarios rather than abstract futures, individuals may overcome motivational barriers and narrow the psycho‐behavioral gaps between awareness and action, either mitigating disengagement or fostering proactive engagement.

### 5.2 When Thinking Hinders Action: Skepticism, Denial and Cognitive Biases

While some disengage due to emotional overload or temporal distance, others do so because of cognitive biases and motivated reasoning.

The international scientific community is unanimous in acknowledging the objectivity and urgent reality of the climate crisis, both with respect to its causes and its tangible effects on people's lives (Cook et al. 2013). However, notwithstanding the abundance of scientific evidence, climate science and engagement with the climate crisis are intertwined with several factors, such as politicization, misinformation, and psychological variables. Together, they could downplay the magnitude of the phenomenon, resulting, for example, in skepticism or denial of the climate crisis.

A key factor in climate skepticism and denial is political alignment (Jylhä et al. 2020; Jylhä and Hellmer 2020). Climate discourse often overlaps with political positions, reflecting a common skeptical stance toward the dissemination of scientific facts by the media, leading to the rejection of climate science and the prevalence of communication strategies about CC that frame the topic as a contentious issue rather than a settled scientific fact (Thapa Magar et al. 2024; Treen et al. 2020).

Notably, denial and other forms of skewed perception of CC are also supported by psychological factors and culturally embedded cognitive biases (Zhao and Luo 2021). Cognitive biases are defined as systematic errors in the way individuals operate rational judgment and make decisions. Biases are driven by heuristics, that is, mental shortcuts that, while simplifying information processing, can lead to systematic errors in perception, reasoning, and behavior (Tversky and Kahneman 1974).

An example is cognitive dissonance (Moser et al. 2022), a mental discomfort experienced in response to conflicting information that makes it easier to reject evidence that conflicts with preexisting beliefs, especially where the conflicting information threatens the status quo (Jylhä and Akrami 2015). Confirmation bias, the tendency to seek and prefer information that confirms preexisting beliefs, also compounds this.

Another critical bias is the availability heuristic and its influence on how people rely on direct and recent personal experience, including that conveyed by the media, to make predictions about climate events. Thus, the result is an unbalanced perception anchored on the availability and nature of available information. Moreover, this tendency can also be combined with the anchoring effect, which is the reliance on the initial information as a reference point for all subsequent reasoning (Visschers 2018).

### 5.3 Coping With Climate Change: From Stress to Adaptive Behaviors

Beyond barriers to engagement, individuals also deploy coping strategies—some maladaptive, others resilient—which interact with emotional and cognitive demands. This section supports the claim that effective climate responses depend on balancing emotional engagement and individual cognitive resources (Klein 2013).

Cognitive sciences could contribute to this by systematically investigating how time cognition, emotional regulation, and decision‐making shape responses to CC at both individual and collective levels. Findings coming from the integrative field of cognitive ecology show that organisms adjust their cognitive capacities—such as memory, problem‐solving, and risk assessment—based on environmental challenges, suggesting that human cognition may similarly evolve to navigate the increasing uncertainties of CC (Mettke‐Hofmann 2014). Furthermore, cognitive neuroscience has lent support to the understanding of how these cognitive abilities unfold at the brain level, also opening the possibility of informing effective interventions in addressing CC. The ability to simulate even distant future events requires resisting the tendency of temporal discounting by exerting cognitive control over the impulse to prefer an immediate reward. These abilities depend on the complementary functionality of the prefrontal cortex (Berkman and Falk 2013) and in particular the dorsolateral portions involved in cognitive control and suppression of short‐term (Peters and Büchel 2010), and ventromedial portions involved in balancing and integrating emotionally relevant elements for the construction of long‐term behavioral responses (Klein 2013). Considering these brain areas as cortical targets for neuromodulation techniques could support individuals in making sustainable lifestyle changes and policy decisions aligned with long‐term environmental goals (Doell et al. 2023; Rae et al. 2022). Although not directly related to CC, emerging evidence shows that the selection of cortical targets such as the dorsolateral prefrontal cortex would produce an enhancement effect of cognitive flexibility, self‐regulation, and resistance to temporal discounting tendency in the context of long‐term decision‐making, essential for climate involvement (Doell et al. 2023). One of the most popular techniques for neuromodulation is transcranial direct current stimulation, which is a noninvasive stimulation technique that interacts with neuronal electrical activity via weak electric currents. Langenbach et al. (2022) provided evidence in favor of the fact that excitatory stimulation of the right temporo‐parietal junction promotes sustainable decision‐making ability in the context of intergenerational mentalizing, that is, the ability to assume the perspective of future generations, even at personal cost. Therefore, the possibility of incorporating neuroscientifically informed interventions in the support of perspective‐taking skills may represent an advancement not only for individual‐level responses but also for long‐term systemic solutions. In the next paragraph, we explore how interdisciplinary dialogue is key for sustainable climate policy.

### 5.4 Toward Interdisciplinary Climate Adaptation: Designing Sustainable Climate Policies

Strategies for adaptation and mitigation of CC cannot only pass through behavioral coping and emission reduction strategies but must also arise from interdisciplinary dialogue capable of impacting people's lives in the contexts in which they operate. This is why environmental psychology and cognitive sciences find a fruitful partner in the architectural sciences, pointing to possible ways of interaction between people's psychological characteristics and their surroundings. In fact, the way environments are built influences cognitive abilities by transcending the limits of aesthetics and logistics to enter fully among the tools that can influence pro‐environmental behaviors (Jeffery 2019). For example, considering projections that by 2050, 68% of the world's population will live in urban areas (UN 2018), it is necessary to take into account that densely populated areas generally tend to discourage long‐term sustainable behaviors by acting on factors such as stress, cognitive load, and decision making (Stier et al. 2021). On the contrary, urban spaces structured around the theme of accessibility through green mobility promote cognitive resilience and influence decision making toward sustainable choices (de Paiva and Jedon 2019). Urban design choices that make it the norm to opt for low‐impact mobility such as public transportation or cycling act on an unconscious level by directing citizens toward sustainable choices, relieving them from the burden of making deliberate environmental choices. Moreover, the design of urban environments that integrate biophilic design and climate‐responsive architecture can act on a cognitive level (Fadda et al. 2023), as well as enhancing psychological well‐being and environmental sustainability by resonating with psychological experiences of emotional attachment to the environment and behaviors of long‐term social protection and engagement (Negrello 2023). These insights resonate with the concept of healing spaces, that is, environments designed to promote emotional, psychological, and cognitive well‐being through access to nature, natural light, reduced noise, and supportive spatial layouts (DuBose et al. 2018). Rooted in the Biophilia Hypothesis (Kellert and Wilson 1995), this approach exploits human innate affinity for natural forms, supporting the claim that exposure to biophilic environments supports restoration, stress reduction, and cognitive functioning. Evidence from healthcare architecture shows that such designs improve not only physical outcomes but also emotional regulation, social connection, and perceived control—key psychological resources for coping with complex stressors like CC (Ulrich et al. 2010). Overall, when faced with the need to imagine uncertain times dominated by the climate crisis, these findings provide evidence that cognitive sciences can enrich the public discourse on the changing future, whether in cities, behavioral interventions, or institutions—and significantly influence individual adaptation and collective resilience. If placed into a fruitful interdisciplinary discourse, they can provide a sensible theoretical framework within which to devise intervention strategies that take into account the psychological and neurocognitive architecture of individuals in order to optimize both psychological well‐being and ecological resilience through a virtuous circle between cognitive adaptive capacities and the emergence of pro‐environmental sustainability behaviors. This opens the way to a broader policy discussion that integrates psychological insights with ethical imperatives such as intergenerational equity, youth participation, and intersectional justice.

### 5.5 Governing the Future: Imperatives for Climate Policy

The long‐term legitimacy and effectiveness of climate policies depend on the ability to gather and give voice to the demands and rights of younger generations. This ethical and social imperative is rooted in intergenerational justice. This concept aligns with the temporal structure of the climate crisis, where the most severe consequences of present inaction will only unfold in decades, affecting younger and future generations. Intergenerational justice requires that the present generation not only avoid passing on the burden of the climate crisis, but also recognize the duty of the current ruling class to safeguard the well‐being and basic freedoms of future generations (Schuppert 2011). Notably, intergenerational justice is no longer just a moral concern: it has proved to be capable of influencing policymakers and draw public attention to the issue of climate protection as an intergenerational responsibility. A pivotal example is represented by the historical decision of the German Constitutional Court in the case *Neubauer et al*. versus *Germany*, recognizing that insufficient climate mitigation violates the constitutional rights of future generations (Steinkamp 2023). This change of pace in the discourse on climate mitigation policies is well aligned with a conception of the climate crisis that requires a shift away from a presentist position focused only on present citizens. Moral and legal devices such as intergenerational justice do in fact respond to the need to consider the temporal dimensions of the problem, solutions, and rights, especially those of younger generations. While younger people are the most vulnerable to the physical and psychological impacts of CC, they are also the most engaged and mobilized for the climate cause. Movements such as *Fridays for Future* combine the need to frame the climate crisis in terms of urgency, justice, and intergenerational responsibility, not only mobilizing mass participation but also altering the elite political discourse, pushing policy makers to acknowledge the diverse dimensions of climate action (Nisbett and Spaiser 2023). Recognizing youth as key actors in climate governance must also account for the psychological and cognitive burdens they disproportionately bear. As shown throughout the section of this review, younger generations face not only future climate risks but present stressors—such as eco‐anxiety, temporal disruption, and cognitive overload—that arise from confronting a changing world. Policymaking frameworks that center youth must therefore be open to intergenerational dialogue and ensure all voices are represented. Therefore, effective and fair climate justice must also be intersectional. Applied to the climate crisis, environmental harms and adaptive capacities are unequally distributed across race, class, gender, age, geography, and ability and could form overlapping systems of oppression. For example, women, Indigenous peoples, and Global South communities often suffer disproportionate climate impacts while remaining underrepresented in policymaking (Tafon and Saunders 2025). Research in intersectional climate justice has demonstrated that adaptation policies often reproduce colonial, gendered, and racialized hierarchies unless explicitly designed to counteract them (Amorim‐Maia et al. 2022). Similarly, community‐based climate adaptation in urban informal settlements is most effective when it addresses historically excluded voices—particularly women and youth—through participatory governance (Rigon 2025).

For policy makers, the path to the future is clearly marked by the need to consider intergenerational equity, intersectional justice, and meaningful youth participation as pillars of climate governance. Therefore, mitigation targets, adaptation strategies, and climate finance mechanisms must be designed not merely with technical feasibility or political convenience, but grounded in ethical commitments to fairness, inclusion, and democratic representation. Without this normative grounding, climate policy risks reproducing or amplifying existing injustices—undermining both its moral authority and its social resilience.

**Neuroarchitecture and Neurourbanism**

Neuroarchitecture and Neurourbanism are research fields emerging from the interdisciplinary exchange between architecture and urbanism and neurocognitive sciences. They are aimed at exploring the bidirectional relationship between the built environment and neurocognitive features, highlighting how spaces influence brain function and how cognition regulates our interaction by shaping perception and behavior (Abbas et al. 2024; Eberhard 2009).

Neuroarchitecture incorporates techniques to visualize brain activity as a function of architectural design, providing empirical insights into design features such as lighting, color, materials, and spatial layouts, accounting for their impact on the neural correlates of processes that regulate our relationship with the environment such as spatial navigation, memory, and emotional regulation (Ghamari et al. 2021).

Neurourbanism extends these elements to urban environments, examining their role in regulating well‐being and mental health (Adli et al. 2017; Senkler et al. 2023). This is accomplished through an urban design capable of limiting the impact of environmental stressors that trigger stress‐related disorders (e.g., sensory overload, high‐density living, and noise) by incorporating restorative elements such as green spaces or wayfinding strategies fostering psychological resilience (Pykett et al. 2020).

Advancements in neuroimaging techniques and biosensing technologies for real‐time physiological monitoring of the interaction with the environment enabled architectural sciences and urbanism to leverage data‐driven neuroscientific insights for designing environments that not only fulfill functional needs but are also attuned to key neurocognitive features. This transformative approach put neuroarchitecture and neurourbanism at the forefront of interdisciplinary research, shaping the future of built environments that enhance holistic well‐being (Ellard 2020).

## 6 Conclusion

Climate change is not merely an environmental crisis but also impacts our existence through the cognitive and emotional constraints that govern how we perceive, process, and make decisions about CC. In this review, we have put forward an interdisciplinary perspective to demonstrate that “temporal cognition” plays a key role in shaping climate change‐related behaviors. The cognitive processes underlying such behaviors are not purely rational but are intertwined with emotions related to the climate crisis and arising from the relationship with time and the awareness that “we are running out of it.” A paradigmatic example of this cognitive‐emotional interplay is “eco‐anxiety,” which can regulate the emotional and behavioral response influencing both engagement and inaction. Therefore, we encouraged shifting the focus toward the cognitive processes involved in how individuals process CC and regulate emotions. We thus provided insight into seemingly paradoxical yet coexisting behaviors oscillating between climate engagement and denial.

Inspired by Maurizio Nannucci's evocative artwork, our work integrates these cognitive insights within an “interdisciplinary dialogue.” This supports the adoption of a nuanced approach that does not simply buffer the negative effects of CC. The inclusion of advancements from related fields such as psychology, neuroscience, and environmental sciences paves the way for a promising direction for improving “long‐term,” “sustainable climate action.” By building on multidisciplinary expertise and centering the lived experiences of those most affected, we can design holistic interventions that not only mitigate the psychological and emotional burden of CC but also advance justice and fuel resilience, ensuring that climate action is both scientifically informed and cognitively sustainable.

## Author Contributions

**Massimo Bertoli:** conceptualization (lead), project administration (equal), supervision (equal), visualization (lead), writing – original draft (lead), writing – review and editing (lead). **Martina De Cesaris:** data curation (supporting), writing – review and editing (supporting). **Sofia Bonventre:** data curation (supporting), writing – review and editing (supporting). **Marcella Brunetti:** conceptualization (equal), project administration (equal), supervision (equal), writing – original draft (supporting), writing – review and editing (supporting).

## Conflicts of Interest

The authors declare no conflicts of interest.

## Related WIREs Articles

The complex act of projecting oneself into the future

Cognitive ecology: ecological factors, life‐styles, and cognition

To do it now or later: The cognitive mechanisms and neural substrates underlying procrastination

It is about time: Conceptual and experimental evaluation of the temporal cognitive mechanisms in mental time travel

## Data Availability

Data sharing is not applicable to this article as no new data were created or analyzed in this study.
